# Striatal Vulnerability in Huntington’s Disease: Neuroprotection Versus Neurotoxicity

**DOI:** 10.3390/brainsci7060063

**Published:** 2017-06-07

**Authors:** Ryoma Morigaki, Satoshi Goto

**Affiliations:** 1 Parkinson’s Disease and Dystonia Research Center, Tokushima University Hospital, Tokushima University, Tokushima 770-8503, Japan; morigakiryoma@hotmail.com; 2 Department of Neurodegenerative Disorders Research, Institute of Biomedical Sciences, Graduate School of Medical Sciences, Tokushima University, Tokushima 770-8503, Japan; 3 Department of Neurosurgery, Institute of Biomedical Sciences, Graduate School of Medical Sciences, Tokushima University, Tokushima 770-8503, Japan

**Keywords:** Huntington’s disease, huntingtin, striatum, medium spiny neuron, pathophysiology, striosome, matrix

## Abstract

Huntington’s disease (HD) is an autosomal dominant neurodegenerative disease caused by the expansion of a CAG trinucleotide repeat encoding an abnormally long polyglutamine tract (PolyQ) in the huntingtin (Htt) protein. In HD, striking neuropathological changes occur in the striatum, including loss of medium spiny neurons and parvalbumin-expressing interneurons accompanied by neurodegeneration of the striosome and matrix compartments, leading to progressive impairment of reasoning, walking and speaking abilities. The precise cause of striatal pathology in HD is still unknown; however, accumulating clinical and experimental evidence suggests multiple plausible pathophysiological mechanisms underlying striatal neurodegeneration in HD. Here, we review and discuss the characteristic neurodegenerative patterns observed in the striatum of HD patients and consider the role of various huntingtin-related and striatum-enriched proteins in neurotoxicity and neuroprotection.

## 1. Introduction

**Figure 1:**
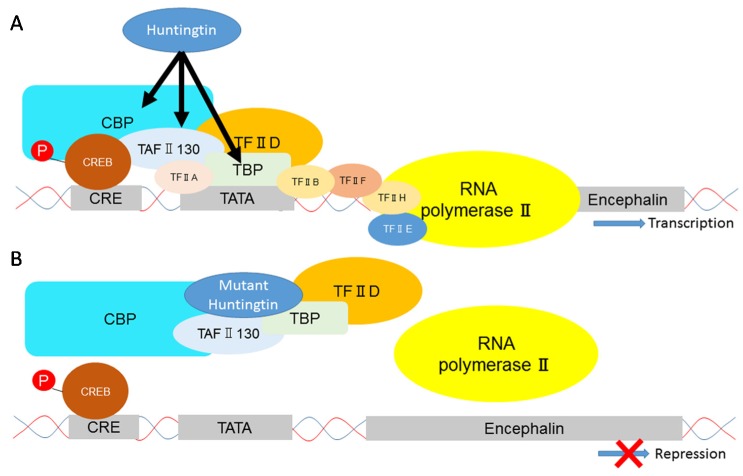
An example of transcriptional dysregulation by mutant Huntingtin protein. (**A**) Wild-type Huntingtin interacts with CREB-binding protein (CBP) and possibly with TATA box binding protein associated factor (TAF) II 130 and/or TATA box binding protein (TBP). Huntingtin promotes transcription of encephalin; (**B**) Mutant Huntingtin binds CBP, TAF II 130 and TBP and prevents these transcription factors from recruitment. P: phosphorylation, TF: transcription factor, CRE: cAMP response element, CREB: cAMP response element-binding protein.

Huntington’s disease (HD) is an autosomal dominantly inherited neurodegenerative disorder characterized by the late onset of gradually worsening motor, cognitive, and psychiatric disturbances [1]. At present, HD is largely untreatable and the mean survival time of individuals with HD is 17–20 years after symptom onset [2]. In HD, a mutation of the protein huntingtin (Htt), which involves the expansion of a cytosine-adenine-guanine (CAG) repeat encoding an extended glutamine tract (PolyQ), causes transcriptional dysregulation (Figure 1) resulting in multiple cellular dysfunctions such as intracellular signaling pathway alterations, protein trafficking defects, synaptic transmission impairments, proteasome dysfunction, and mitochondrial alterations [3,4,5]. Htt is highly conserved among vertebrates and is expressed ubiquitously in the human body [6]. Its highest levels are found in the brain, where it is expressed in all neurons and glial cells [3]. Despite the ubiquitous expression of mutant Htt (mHtt) throughout the brain, human pathology has shown that degeneration is specific to certain neuronal subpopulations affecting the striatum and, to a lesser extent, the cerebral cortex in patients with HD [4]. In the striatum, a differential involvement of striosome and matrix compartments has been reported in HD [5,6,7,8,9,10,11,12]. Moreover, ongoing neurodegeneration is preferentially found in medium spiny neurons (MSNs) and parvalbumin-expressing interneurons in the striatum [13,14,15,16,17,18]. These findings indicate that striatal cell type- and compartment-specific vulnerabilities may underlie the etiology of striatal pathology in HD [19]. Here, we discuss striatal cell type- and compartment-specific degeneration in HD as well as neuroprotection and neurotoxicity associated with Htt-related and striatum-enriched proteins.

## 2. Striatal Anatomy

**Figure 2:**
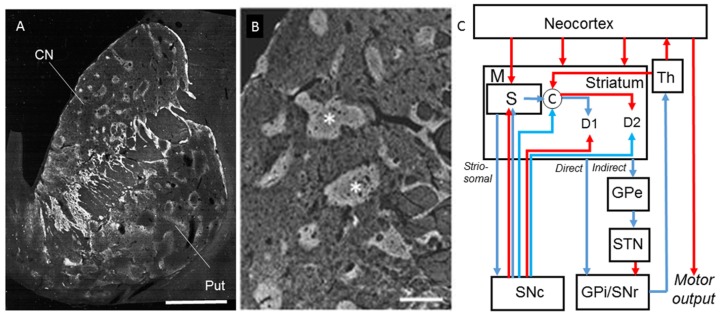
Striatal structural anatomy and functional circuitry model. (**A**) A human striatum section immunostained with anti-Met-enkephalin antibody. (From Goto et al. (2015) with permission) [29]; (**B**) Low-power-magnified microscopic negative image of the caudate nucleus. Asterisks indicate striosomes (From Goto et al. (2015) with permission) [29]; (**C**) Striatum plays a central role of multiple feedback and feedforward regulations in motor processing. Connectivity diagram showing excitatory pathways as red and inhibitory pathways as blue. Scale bars: (**A**) 4 mm; (**B**) 1 mm. CN, caudate nucleus; Put, putamen; S, striosomes; M, matrix; C, Cholinergic interneurons; D_1_, medium spiny projection neurons expressing dopamine D_1_ receptors; D_2_, medium spiny projection neurons expressing dopamine D_2_ receptors; SNc, substantia nigra compact; GPi, globus pallidus internus; SNr, substantia nigra reticulata; STN, subthalamic nucleus; GPe, glubus pallidum externus; Th, thalamus.

In HD, the striatum has been identified as the primarily affected structure, which undergoes severe degeneration. As a core structure of basal ganglia circuits, the striatum integrates midbrain dopaminergic inputs and neocortical and thalamic glutamatergic inputs, and then sends GABAergic outputs to its target nuclei, such as the globus pallidus and substantia nigra. The striatum plays a critical role in processing information related to motor function and reward- and goal-oriented behavior. To facilitate the understanding of HD neuropathology, we briefly review normal striatal anatomy (Figure 2).

### 2.1. Fundamental Structure

Respectively, MSNs (10–24 μm in diameter) and medium aspiny interneurons represent approximately 90% and 10% of striatal neurons in rodents, and approximately 75% and 25% of striatal neurons in primates [20,21]. The four GABAergic interneuron subtypes found within the striatum express (1) parvalbumin; (2) calretinin; (3) coexpressing nicotinamide adenine dinucleotide phosphate (NADPH) diaphorase, nitric oxide synthase (NOS), neuropeptide Y (NPY) and somatostatin; and (4) tyrosine hydroxylase (TH) expressing interneurons [20,21,22,23,24]. Parvalbumin-positive interneurons (i.e., “fast-firing interneurons”) can block or delay the firing of more than 100 MSNs [22,25]. NOS-containing interneurons (i.e., “low-threshold spike interneurons”) release nitric oxide (NO) via dopamine D_1_/D_5_ receptor (D_1_R/D_5_R) activation and influence the induction of long-term depression (LTD) via the cyclic guanosine monophosphate (cGMP) pathway [26]. Large aspiny cholinergic interneurons (21–45 μm in diameter) represent 1%–2% of the total cell population in the human striatum [27]. The cholinergic interneurons play a role in the spatiotemporal selection of convergent inputs to striatal MSNs [28].

Striatal MSNs send inhibitory GABAergic efferents to their target brain regions. Approximately half of MSNs project via the “direct pathway” to neurons within the internal globus pallidus (GPi; i.e., the entopeduncular nucleus in some species) and substantia nigra pars reticulata (SNr) [30]. The rest of the MSNs project via the “indirect pathway” to neurons within the external globus pallidus (GPe) [30]. Direct pathway MSNs express the D_1_R subtype and muscarinic M4 receptors and contain the neurotransmitters dynorphin and substance P. Indirect pathway MSNs express the dopamine D_2_ receptor (D_2_R) subtype and adenosine A_2A_ receptors (A_2A_R) and contain enkephalin [31,32]. Activation of D_1_R or A_2A_R increases cyclic adenosine monophosphate (cAMP)/protein kinase A (PKA) signaling, whereas activation of D_2_R decreases it. Phosphodiesterase 10A (PDE10A) inhibitors decrease cAMP/PKA signaling in direct and indirect pathway MSNs [33,34]. The direct and indirect pathways converge at the GPi/SNr complex, which then transmits its GABAergic outputs to the thalamocortical pathway and brainstem locomotor region, including the pedunculopontine nucleus (PPN) [30]. MSNs receive excitatory glutamatergic inputs originating from the neocortex (i.e., layers III and V) and thalamus (i.e., mainly from the intralaminar thalamic nuclei). MSNs possess 10,000–15,000 spines; however, corticostriatal terminals form a small number of synapses with individual MSNs. This indicates that a massive convergence of cortical inputs may occur at this level [22,35]. Synaptic convergence may also apply to thalamic inputs since the number of thalamostriatal and corticostriatal synapses within the striatum is of a similar magnitude [35]. Striatal GABAergic interneurons receive cortical and thalamic excitatory inputs and provide strong feedforward inhibition (i.e., inhibitory postsynaptic potentials (IPSPs)) to the proximal dendrites of MSNs [22,36]. This feedforward inhibition is more powerful than the reciprocal feedback inhibition produced by MSN axons [28].

### 2.2. Striatal Compartments

The striatum encompasses a three-dimensional labyrinthine structure composed of striosome and matrix compartments [37,38,39]. Across mammalian species, striosomes occupy 10%–20% of the striatum [40,41,42]. Striosomes are organized early in embryonic development and receive developmental cues from dopaminergic inputs originating in substantia nigra pars compacta (SNc) [43,44,45]. The striosomal and matrix compartments have MSNs containing D_1_R and D_2_R subtypes. In primates, D_1_Rs are predominantly localized in striosomes, whereas D_2_Rs are localized in the matrix [38,46]. Striosomal MSNs project to the ventral tier of the SNc or its immediate environs [47,48,49]. Striosomal MSNs also innervate the habenula, which projects to SNc dopaminergic neurons [50,51,52,53,54]. Reciprocal striosome-SNc innervation strongly regulates nigral dopaminergic neurons, thereby maintaining critical control over dopaminergic actions within the striatum [38,55]. Different cortical regions innervate the striosome and matrix compartments. The prelimbic, infralimbic, caudal orbitofrontal, and pregenual anterior cingulate cortices innervate striosomes, whereas sensorimotor cortices innervate the matrix [56,57,58,59,60,61,62,63]. These input fibers as well as striatal MSN dendrites and axons mostly remain within each compartment [64,65]. Large cholinergic interneuron cell bodies are located in the matrix at the edge of the striosome-matrix boundary [66,67,68,69,70,71]. The dendrites of cholinergic interneurons spread to both compartments, whereas their axons are densely distributed within the matrix. Cholinergic interneurons are thought to mediate inter-compartmental information processing [66,67,70,71,72]. Neurons of the centromedian-parafascicular thalamic nuclei innervate and modulate striatal cholinergic interneurons [73].

It is known that reward-related, limbic-based circuits and sensorimotor and associative circuits are concentrated in the striosome and matrix compartments, respectively [70]. However, differential activity in the striosomes possibly produces distinct reinforcement-related signals, which give rise to repetitive behaviors [74,75]. Moreover, the existence of a direct, reciprocal circuit between striosomes and dopamine-containing SNc neurons suggests that striosomes affect dopamine release within the matrix through a negative feedback mechanism [19,76,77,78,79]. An anatomically- and physiologically-based computational model of reinforcement learning revealed that the striosomes and matrix are responsible for motor focusing and scaling, respectively [72].

Striosomal MSNs receive cortical input from the infralimbic cortex, which evaluates and controls ongoing behaviors [80]. This evaluation cortex sends signals related to responsibility of selected modules to striosomes. Positive or negative responsible signals are conveyed from striosome into matrix via cholinergic or, possibly, palvalbumine interneurons [72]. Striosomal MSNs of the striatonigral pathway release substance P, which depolarizes cholinergic interneurons and induces acetylcholine release. The released acetylcholine excites matrix interneurons via nicotine receptors and inhibits matrix MSNs. Striosomal MSNs of the striatofugal pathway release enkephalin, which either directly or via μ-opioid receptors (MORs) activation, hyperpolarizes cholinergic interneurons in the matrix. Thus, activation of striosomal MSNs results in the inhibition of acetylcholine release and subsequent disinhibition of matrix MSNs [70,81]. Cholinergic interneurons are called tonically active neurons (TANs), and always release acetylcholine into the striatum as background activity [71]. During sensorimotor learning, dopamine neurons in the SNc exhibit rapid and brief bursts of activity that coincide with TAN pause phase in response to a conditioned stimulus which predicts reward. TAN pause response amplifies the release of dopamine only in the targeted area in the matrix compartment [82,83]. The activation of dopamine receptors is required for spike timing-dependent long-term potentiation (LTP) at striatonigral direct-pathway MSNs and LTD at striatopallidal indirect pathway MSNs in the matrix compartment [84,85,86]. TAN pause exerts opposing plasticity effects to these two pathways in order to enhance the disinhibition of actions by direct-pathway MSNs and to reduce the inhibition of actions by indirect-pathway MSNs [86]. Thus, the striosomal activation and deactivation in selective modules might not only enhance the contrasts between modules but serve to limit the spatial extent of responsibility signaling via a surround inhibition-like mechanism [83]. Dopamine release in the matrix decides the scaling of the selected modules. Activation of striosomal MSNs inhibits dopamine-containing SNc neurons and terminates goal-directed behavior by reducing dopamine release in the matrix [70].

Imbalances in the activity of the striosome and matrix compartments could produce changes in the selection and release of motor and behavioral functions alternatives via inter-compartmental or striatonigral reciprocal pathways [19,72,74,87]. According to this “compartment” hypothesis, the correlation between specific clinical symptoms and the activity of striatal compartments is important for understanding basal ganglia disorders, such as HD and dystonia [19,74,79,88,89].

## 3. Striatal Pathology

HD represents one of the main classes of basal ganglia disorders, as well as Parkinson’s disease and dystonias. Accumulating evidence suggests that the basal ganglia circuit architecture participates in the creation of striatal pathology in HD [5,6,7,8,9,10,11,12,89]. Considering the recent advances in our understanding of the anatomy and functional roles of the striatum, we discuss cell type- and compartment-specific striatal vulnerabilities in HD (Table 1).

### 3.1. Wild-Type and mHtt Interacting Proteins

Wild-type Htt, a 347-kDa protein with multiple scaffolds, acts as a major protein interaction hub and an orchestrator of converging intracellular trafficking and signaling pathways [90,91]. Wild-type Htt has anti-apoptotic properties against starvation, mitochondrial toxins, and mHtt overexpression [92,93,94] and is essential for normal embryonic development [95,96,97]. The anti-apoptotic effects of wild-type Htt may be associated with the inhibition of pro-apoptotic protein caspase-3 or pro-caspase-8 through the sequestration of pro-apoptotic protein huntingtin-interacting protein 1 (HIP1) and HIP1 protein interactor (HIPPI) [98,99]. Wild-type Htt also acts as a positive transcriptional regulator of neuron-restrictive silencer element (NRSE)-regulated genes, such as brain-derived neurotrophic factor (BDNF) [100,101]. Moreover, wild-type Htt interacts with microtubule-based motor complex-related proteins (i.e., dynein/dynactin and kinesin), which are essential for the axonal transport of vesicles [101,102,103,104,105]. Decreased BDNF levels have been demonstrated in cellular and animal models of HD and patients with HD [106]. A quantitative proteomic analysis revealed that HD pathogenesis may be linked to changes in Htt interactions with stress granule-associated RNA-binding proteins (i.e., cytoplasmic activation- and proliferation-associated protein 1 (Caprin-1) and GTPase-activating protein Src homology 3 (SH3) domain-binding protein 1 (G3BP-1)) [91]. The Caprin-1/G3BP-1 complex regulates the transport and translation of mRNAs of proteins associated with neuronal synaptic plasticity, including BDNF [91]. Although BDNF deletion mutant mice had selective loss of olfactory bulb parvalbumin-containing interneurons via the phospholipase C gamma (PLCγ) pathway, other calcium-binding, protein-containing neuron populations were unaffected [107].

Autosomal dominant inheritance and other genetic studies strongly indicate that polyglutamine (polyQ) expansion confers a toxic gain of function to Htt [108,109,110]. mHtt protein is cleaved by caspases, calpain, and aspartic endopeptidase, and N-terminal fragments containing the polyQ expansion are sufficient to produce HD-like abnormal clinical syndromes and intranuclear inclusions in HD animal models [111,112,113]. In mice, inhibition of caspase-6-dependent cleavage retains full-length mHtt, which prevents behavioral and neuropathological dysfunction [114]. Thus, the cleavage of benign full-length mutant huntingtin into toxic fragments may be a rate-limiting step in HD pathogenesis [90]. The proteolysis and subsequent toxicity of mHtt can also be suppressed with the phosphorylation of Htt by protein kinase B (Akt), cyclin-dependent kinase 5 (Cdk5), and extracellular signal-regulated kinase 1 (ERK1) [90,115,116,117].

mHtt induces mitochondrial dysfunction by reducing adenosine triphosphate (ATP) generation [118], calcium buffering [119,120], and mitochondrial trafficking [121,122]. A quantitative proteomic analysis revealed that the most altered interactions occur between Htt and several mitochondrial proteins, including apoptosis inducing factor, mitochondria associated 1 (AIFM1) [91]. MSNs usually maintain electrophysiologically low levels of spontaneous discharge, which require a large amount of ATP for the maintenance of a hyperpolarized state [123]. This mechanism may underlie the vulnerability of MSNs to mitochondrial dysfunction [110].

Some authors have reported a “dying-back” pattern of neuronal degeneration in HD, which suggests that deficits in axonal transport underlie the increased vulnerability of projection neurons to mHtt [108,124,125]. mHtt inhibits axonal transport through the activation of the c-Jun N-terminal kinase (JNK) pathway and phosphorylation of molecular motor proteins [126].

Wild-type Htt and mHtt interact with transcriptional factors, including cAMP response element-binding (CREB)-binding protein (CBP), TATA-binding protein (TBP), p53, specificity protein 1 (Sp1), transcriptional repressor element-1 transcription factor/neuron restrictive silencer factor (REST/NRSF), TAF II 130, and peroxisome proliferator activated receptor γ coactivator 1α (PGC-1α) [127]. However, these proteins are diffusely expressed throughout the brain and do not appear to explain cell type- or compartment-specific neuronal degeneration.

### 3.2. Positron Emission Tomography (PET) Imaging Studies

PET imaging studies provide important insights about HD pathogenesis. PET can detect various molecular changes in HD gene expansion carriers before disease manifestation [128,129]. Currently, one of the most important biomarkers for patients with HD is PDE10A, which is highly expressed in MSNs, but not in interneurons [33,130,131,132,133]. PDE10A is a dual substrate that regulates cAMP/PKA signaling and hydrolyzes cAMP and cGMP with an approximately 20-fold higher affinity for cAMP [134]. The inhibition of PDE10A activates cAMP/PKA signaling within direct- and indirect-pathway neurons [34]. Previous PET studies have shown that PDE10A expression decreased in the striatum and pallidum and increased in the motor thalamus of premanifest HD gene carriers compared to matched healthy controls [135]. PET imaging research using [^11^C] IMA107 demonstrated a 25%–33% reduction in striatal PDE10A 25 years earlier than predicted symptomatic onset [135,136]. Decreased PDE10A expression was mostly restricted to the dorsal sensorimotor striatum [135], and lower striatal PDE10A expression was associated with disease burden and severity [137]. The PDE10A signal decreased by 33%–34% in the striatum and increased by 35% in the motor thalamus [135]. In HD animal models, PDE10A inhibition reduced neurodegeneration in striatal and cortical neurons and delayed neurological deficit development [138,139]. PDE10A inhibition causes an up-regulation of cAMP/PKA signaling and CREB phosphorylation and increases BDNF expression in striatal neurons [138]. Moreover, PDE10A inhibition has a greater facilitatory effect on the corticostriatal synaptic activity of indirect-pathway neurons than on that of direct-pathway neurons [140].

Increased microglial activation begins at approximately 15–20 years before symptom manifestation [141,142,143]. Moreover, several studies have reported decreases in D_2_Rs, brain metabolism, and cortical gray and white matter volume at approximately 10 years before disease manifestation [128,129]. In premanifest HD gene carriers, D_1_Rs and D_2_Rs are significantly reduced by 25%–50% [144,145]. At the premanifest stage, the mean annual decline rate is 2% for D_1_Rs and 4%–6.3% for D_2_Rs [144,146].

### 3.3. Striatum-Predominant Neurodegeneration in HD

Along the sagittal axis of the brain, the caudal caudate nucleus (CN) and putamen show greater neuronal loss than the rostral CN and putamen. Moreover, along the coronal axis of the brain, the dorsal and medial striatum are more degenerated than the ventral and lateral striatum [3]. Magnetic resonance imaging (MRI) studies have shown the same striatal atrophy gradients [147,148]. In the early stages of HD when chorea is most apparent, there is a preferential loss of neurons from the indirect pathway projecting to the GPe [13]. The increased frequency of movement release associated with the early and selective involvement of indirect-pathway neurons may explain the genesis of chorea in HD. This notion is supported by evidence obtained from a transgenic mouse model study in which indirect-pathway neurons bearing D_2_Rs were selectively ablated [149].

Wild-type Htt and mHtt are expressed in the brain without significant inter-regional variation, which suggests that the pathogenic process is not a direct effect of mHtt toxicity [150,151]. Increased glutamine tract lengths of mHtt are more prominent in the striatum than in the cortex [152,153]. Although striatal vulnerability is correlated with the size of the CAG repeat expansion in the Htt located on the short arm of chromosome 4 [154], the mechanisms causing this increasing vulnerability of the striatum are yet to be elucidated [3].

In patients with HD, striatal vulnerability may be associated with reduced corticostriatal input due to impairment of BDNF, an important neuromodulator and trophic factor. Wild-type Htt positively regulates BDNF, whereas mHtt fails to regulate BDNF [155]. BDNF is reduced in the brains of mice and humans with HD. This reduction in BDNF may be attributable to mHtt-induced reduction of anterograde axonal transport within cortical neurons [101,103,156], decreased BDNF endocytosis by MSNs [157], or both of these mechanisms [110]. BDNF supports MSN survival and is required for the dendritic growth of striatal neurons [106,158]. Interestingly, decreased BDNF levels induce selective neuronal degeneration in enkephalinergic, indirect-pathway MSNs [159].

Rhes is a guanine nucleotide-binding protein that may affect small ubiquitin-like modifier (SUMO) modification and preferentially interact with mHtt [160]. Rhes is predominantly expressed in the striatum and, to lesser extent, in other forebrain areas affected by HD. Htt and Rhes interactions may underlie the regional specificity of HD [160,161]. Rhes binds to mHtt and acts as a SUMO E3 ligase to stimulate sumoylation of mHtt, which increases mHtt toxicity [160,162]. In a toxin model of HD, the deletion of Rhes dramatically reduced striatal degeneration and motor dysfunction [163,164]. Moreover, Rhes normally binds to and activates the mechanistic target of rapamycin (mTOR), which inhibits autophagy (i.e., lysosomal degradation); however, in cells with robust autophagy, Rhes activates autophagy via inhibitory binding of Bcl-2 to Beclin-1 [165]. Proteasomal degradation of mHtt prevents cytotoxicity early in life; however, when proteasomal function is compromised by normal aging, autophagy is required. Thus, functional changes associated with aging may explain delayed symptom onset in HD [165,166]. In the striatum, Rhes and mHtt interactions augment cytotoxicity and diminish the autophagic capacity of the neuron [165]. On the other hand, other authors postulate that Rhes is a neuroprotective protein against mHtt-induced neuronal cell death. Rhes levels are reduced in HD patient caudate nucleus and HD mouse model striatum [167,168]. siRNA knockdown of Rhes exacerbates striatal atrophy and behavioral phenotypes in transgenic HD mice [169]. In addition, restoring Rhes alleviates motor deficits and brain pathology in HD mice by activating autophagy of mHtt via increasing Beclin-1, and by altering mTORC1-induced gene expressions implicated in promoting mHtt degradation [168]. mHtt binds both Rhes and mTOR, which reduces the available level of these proteins for intact signaling. Concomitant loss of Rhes and mTOR may render the striatum more vulnerable to early degeneration in HD [168].

### 3.4. Cell Type-Specific Vulnerability

In HD, the most striking neuropathology is the primary and progressive degeneration of MSNs and parvalbumin interneurons and relative sparing of cholinergic and NOS-containing interneurons in the striatum [12,13,14,18,21,170,171]. Htt and its mRNA are widely expressed in the brain; however, they are less abundant in the striatum compared to many brain regions [150,172,173,174,175]. In HD, the amount of Htt and its mRNA present in surviving striatal neurons is not obviously altered, which suggests that Htt expression is not associated with cell type-specific loss in the striatum [175,176,177,178,179]. Moreover, mHtt overexpression may promote functional abnormalities in other neurons besides MSNs, which suggests that MSN vulnerability is not specific to mHtt [180,181].

#### 3.4.1. Glutamate Excitotoxity

In HD, glutamate excitotoxicity within the striatum has been proposed as a possible cause of MSN vulnerability [182,183,184,185,186,187]. Cell type-specific loss of neurons is a hallmark of striatal excitotoxic lesions [188]. Previous studies have shown a reduction of *N*-methyl-d-aspartate (NMDA) and α-amino-3-hydroxy-5-methyl-4-isoxazolepropionic acid (AMPA) receptors in the CN and cerebral cortex of symptomatic patients [189,190] and animal models [191,192] with HD. In animal models of HD, decrease of striatal glutamate receptors and glutamate release has been reported [193,194]. In contrast to symptomatic HD models, AMPA and NMDA currents in pre-symptomatic animal models of HD increased only during the early stages and decreased during a later stage [195,196,197].

NMDA glutamate receptors form heteromeric dimers of NR1 and NR2 subunits [198]. The NR2B and NR2D subunits are highly expressed in MSNs and striatal interneurons, respectively [199,200]. NR1 subunits are essential for NMDA receptor function. When NR1 is heteromerized with NR2, the permeability of the NMDA channel increases over 100-fold and deactivation time also increases [201]. This differential expression pattern may explain the increased vulnerability of MSNs [110]. The balance between synaptic and extrasynaptic NMDA receptors activity may underlie the determination in neuronal cell survival in HD [202,203,204,205]. Synaptic NMDA receptor activity promotes formation of nontoxic mHtt aggregation [202]. Increased NR2B subunit containing extrasynaptic NMDA receptor expression, and current and associated reduction in CREB activation in HD mouse striatum, correlate with mutation severity [203,204]. Activation of extrasynaptic NMDA receptors increases the soluble and toxic mHtt, in part by increasing Rhes expression [202]. Increased toxic mHtt binds to CBP and causes transcriptional deregulation of the CREB-PGC-1α cascade [202]. NR2B subunit containing extrasynaptic NMDA receptors also cause dysregulation in p38 MAPK and CREB signaling in HD model mice [205]. Calabresi et al. reported that MSNs were more sensitive than cholinergic interneurons to group I metabotropic glutamate receptor agonists and ionotropic glutamate receptor agonists, such as kainite, AMPA, and NMDA [184,185]. Moreover, in HD, increased expression of the calcium-binding proteins parvalbumin and calretinin is positively associated with interneuron survival [206]. Parvalbumin and calretinin exert calcium-buffering effects in response to excessive calcium-induced excitotoxicity and are thought to be neuroprotective and important for the survival of interneurons [207,208]. However, the enrichment of calcium-permeable AMPA receptors in parvalbumin interneurons may be a potential pathogenic mechanism [209]. Optineurin is preferentially distributed in interneurons within the naïve mouse striatum [210]. Through its interactions with postsynaptic density protein 95 (PSD-95) or optineurin, wild-type Htt becomes a negative regulator of glutamate receptors [211,212]. This indicates that, with PSD-95 or optineurin, wild-type Htt could exert neuroprotective effects against excessive glutamatergic input [210].

#### 3.4.2. Mitochondrial Dysfunction

In post-mortem studies of HD brain tissue, decreased activity in mitochondrial respiratory chain complexes II, III, and IV was found [213]. By maintaining a hyperpolarized state requiring high amounts of energy, striatal MSNs usually remain electrophysiologically silent [123,214]. The unique energy requirements of MSNs may be associated with their susceptibility to mitochondrial dysfunction [215]. Cholinergic interneurons are enriched in the superoxide free radical scavengers superoxide dismutase 1 and 2 (SOD1 and SOD2), whereas MSNs contain low levels of these enzymes [216]. Although oxidative damage is rarely reported in early-stage HD, it may be a major mechanism during later disease progression [217].

### 3.5. Striatal Compartment-Specific Degeneration

#### 3.5.1. Striosome vs. Matrix Neurodegeneration

Differential neurodegeneration of the striatal compartments has been implicated in HD [5,6,7,8,9,10,11,218]. In the early stage of the disease, a preferential loss of striosomal neurons that gradually spreads to matrix neurons has been reported [5,6,7,8,9]. However, some studies reported preferential loss of matrix neurons [9,10,11,12]. Tippet et al. demonstrated that HD cases with pronounced striosomal neuron loss had shorter CAG repeat lengths than cases with matrix neuron loss or mixed compartmental loss [9]. Moreover, individuals with pronounced striosomal neuron loss did not die during the early stages of disease progression and exhibited more severe mood disturbances [9]. There is a strong inverse relationship between mHtt CAG repeat size and age of onset [151,219] and death [220]. However, disease duration does not vary between individuals with short or long CAG repeats [220]. These results potentially indicate that individuals with preferential matrix neuron loss have relatively rapid disease progression. The preferential neurodegeneration of striosomal MSNs has been reported in HD, as in cerebral ischemia and X-linked dystonia-parkinsonism, the other transcriptional dysregulation syndrome [8,79,184,188,221]. The preferential loss of MSNs in the striosome compartment relative to those in the matrix compartment is thought to be an important factor in the development of abnormal involuntary movements (e.g., dystonias) [19,79,88,188,222]. Tippet found that, in cases with preferential striosome or matrix neurodegeneration, there was no difference in the severity of motor disturbance, even at the end stage of the illness [9]. Neurons within the matrix compartment project from the striatum to the basal ganglia circuitry and are responsible for motor scaling [72]. Consequently, instead of producing chorea, matrix neurodegeneration only induces multiple system atrophy of the parkinsonian type [223]. The striosome compartment is organized early in embryonic development and occupies 10%–20% of the striatum across mammalian species [40,41,42]. If striosomal neurodegeneration reflects the D_1_R decrease observed in striatal PET studies, the annual striosomal degeneration rate is estimated to exceed that of D_2_Rs at the premanifest stage by 2–3-fold. We hypothesize that individuals with pronounced matrix neurodegeneration had prior striosomal neurodegeneration to some extent, and rapid matrix neuron loss may produce the matrix-predominant neurodegeneration pattern. Since the striosome and matrix compartments are responsible for motor focusing and motor scaling [72], striosomal neurodegeneration is sufficient to induce abnormal movements. Matrix D_1_Rs are required for the maintenance of exaggerated movements, and neurodegeneration of matrix D_2_Rs reinforces this enlargement in movements as well. Rapid matrix neurodegeneration may be associated with mHtt-related neurotoxicity. Since autopsied brains are usually acquired at the end stage of the disease, it may be useful to investigate the striatal neuropathology of animal models. In a study of a transgenic rodent model of HD, preferential loss of striosomal neurons was reported [224]. Moreover, a PET study of early premanifest HD gene carriers revealed that extra-striatal PDE10A expression decreased by 25% and 50% in the insular cortex and occipital fusiform gyrus, respectively [136]. These cortical areas are associated with cognitive and limbic functions and are striosome-related areas [225].

#### 3.5.2. Dopamine Excitotoxicity-Induced Striosomal Cell Vulnerability

Preferential striosomal neurodegeneration may disinhibit dopaminergic neurons in the SNc via the striosomal pathway [19]. It is hypothesized that, in early-stage HD, striatal dopamine levels are up-regulated, which induces hyperkinetic movement disorder and striatal dopamine excitotoxicity [226]. This hypothesis is supported by evidence that anti-dopaminergic agents are effective suppressants of abnormal hyperkinetic movements in patients with HD [227]. A study quantifying dopamine and dopamine metabolite levels in autopsied HD human brains found increased dopamine levels in the striatum and substantia nigra [228]. These results were corroborated by a study that found increased dopamine metabolite levels in the cerebrospinal fluid of patients with HD [229]. TH, the rate-limiting enzyme for catecholamine synthesis, is highly concentrated in the neostriatum [230]. In HD, increased TH activity corresponds to increased cellular dopamine levels and neurotoxicity [231,232]. In a study of R2/6 transgenic mice, TH activity increased during early-stage HD and significantly decreased at a later stage [230]. In another previous study, dopamine levels in the CN and putamen of patients with HD were normal [233]; however, since striatal D_1_Rs and D_2_Rs were decreased, a relative increase in dopamine transmission could occur at the network level. Dopamine overflow in the striatum also results in a relative increase in striatal glutamatergic inputs.

Preferential striosomal MSN degeneration may disrupt motor focusing via the acetylcholine-mediated transcompartmental pathway and increase motor scaling via increased striosomal pathway-mediated dopaminergic input [72]. In HD, excessive activity of the dopamine and glutamate pathways may exert neurotoxic effects on the striatum [161,227]. Generally, D_1_R overstimulation exerts a neurotoxic effect, whereas D_2_R stimulation can be neuroprotective [234]. The D_1_R and G-protein α subunit (Gαolf) are preferentially expressed in the striosome compartment, which may be associated with striosomal vulnerability [87,235]. Rhes normally reduces agonist-stimulated cAMP by binding to Gαi [236,237] or inhibiting Gs/olf-mediated signaling [237,238,239,240]. However, Rhes is not involved in the D_2_R/Gαi–mediated adenylyl cyclase inhibition and does not directly interact with D_1_R [237]. Recent study suggests that decreased level of Rhes increases A_2A_R/cAMP/PKA activity selectively under the conditions of dopamine/adenosine-related drug challenge in A_2A_R/D_2_R-expressing MSNs [240]. A_2A_R is evenly distributed in the striatum [87], hence preferential striosomal expression of Gαolf may decide excitatory neurotoxic effects of A_2A_R/cAMP/PKA signaling in A_2A_R/D_2_R-expressing MSNs [241].

#### 3.5.3. The NPY System Exerts Protective Effects on the Matrix Compartment in HD

In HD, early-stage preferential striosomal neurodegeneration is followed by late-stage neuron loss in the surrounding matrix [5,6,7,8,10,79,188]. In patients with HD, the number of NPY-positive cells increases in the striatum [188,242]. NPY exerts an inhibitory effect on glutamate release and microglial activation [243]. Interestingly, NPY fibers are largely distributed in the matrix compartment of the striatum. This suggests that NPY exerts greater neuroprotective effects against excitotoxicity induced by excessive glutamate and microglial activation in the matrix compared to the striosomes [188].

#### 3.5.4. Other Proteins May Underlie the Differential Excitotoxicity between Striosome and Matrix Compartments

Enhanced dopaminergic and glutamatergic inputs to the striatum may cause neurotoxicity due to impaired calcium-buffering capacity and subsequent neurodegeneration of the matrix compartment [225,244,245,246]. In HD, increased expression of calcium-binding protein calbindin-D28K is positively associated with interneuron survival [206]. Calbindin-D28K is predominantly found in the matrix and thought to exert neuroprotective effects that promote calcium-buffering in response to excessive calcium-induced excitotoxicity [207,208]. Crittenden et al. found that calcium diacylglycerol guanine nucleotide exchange factors (CalDAG-GEFs), which are striatum-enriched calcium and diacylglycerol binding proteins, are severely down-regulated in the R6/2 mouse model of HD and post-mortem striatal tissues from patients with HD [89]. Knockdown of matrix-predominant CalDAG-GEF1 protein expression protects against the deleterious effects of mHtt overexpression and may be a compensatory response to MSN vulnerability to mHtt expression [89]. Media et al. reported lower SOD2 expression levels in the striosomal compartment compared to the matrix compartment. This may be associated with preferential striosomal vulnerability to oxidative stress-induced free radical generation [216].

Wild-type Htt is a negative regulator of glutamate receptor and D_1_R activities via interaction with PSD-95 [211]. This indicates that wild-type Htt and PSD-95 could exert neuroprotective effects against excessive glutamatergic and dopaminergic inputs. PSD-95 not only negatively regulates NMDA glutamate signaling, but also dopamine D_1_ signaling at post-synaptic transmission sites. These PSD-95 activities may also exert protective effects against excessive glutamatergic and dopaminergic inputs in the matrix compartment [247]. PSD-95 is predominantly distributed in the matrix relative to the striosomes, which suggests that striosomes are more vulnerable to glutamatergic or dopaminergic excitotoxicity [247]. By phosphorylating dopamine and cAMP-dependent protein kinase, Cdk5 also acts as a negative regulator of postsynaptic dopaminergic signaling [248] and may exert protective effects against excessive dopamine. Activated Cdk5 (i.e., Cdk5 with phosphorylation at the tyrosine 15 residue) is a matrix-enriched protein and may exert neuroprotective effects within the matrix compartment [249].

## 4. Conclusions

When examining pathophysiological changes in patients with Huntington’s disease, several factors should be taken into consideration. For several reasons, in early-stage HD, preferential striatal MSN neurodegeneration and predominant striosomal neuron loss occur due to susceptibility, not specificity. This implies that it is important to examine the differences between MSNs and non-MSN neurons or between the striosomal and matrix compartments of the striatum. Structure-specific protein expression may be a key contributor to the neurodegenerative phenomena of the HD brain (Table 1). These proteins exert neurotoxic or neuroprotective functions against oxidative stress, glutamatergic or dopaminergic excitotoxicity, and dysregulation in autophagy or axonal transport. MSNs are enriched in NR2B and deficient in optineurin, which may explain their susceptibility to glutamatergic excitotoxicity. Moreover, the higher energy requirements and low SOD expression of MSNs may be associated with their vulnerability to mitochondrial dysfunction. Striosomes are enriched in D_1_R and Gαolf and deficient in calbindin-D28K, PSD-95, NPY, and CDK5-pY15. Consequently, striosomal MSNs may be more susceptible to D_1_R- and glutamate-mediated excitotoxicity than matrix MSNs. A better understanding of the pathogenic mechanisms by which neurodegeneration primarily and progressively occurs in the striatum in HD patients can be achieved with further in vivo and in vitro studies on striatal cell type- and compartment-specific vulnerability to neurotoxicity caused by mHtt.

## Figures and Tables

**Table 1:** Neuroprotective or neurotoxic effects of structure-specific proteins.

Affected Structures or Cell-Types	Neuroprotective or Neurotoxic Effects	Factors	Hypothesized Mechanism
Striatum	Neurotoxic or neuroprotective	Predominant expression of Ras homolog enriched in striatum (Rhes) in the striatum	Neurotoxic: Rhes binds mHtt and increases cytotoxicity or decreases the autophagic capacity of the striatal neurons.
			Neuroprotective: Rhes activates autophagy of mHtt and induces gene expression promoting mHtt degradation.
MSNs	Neurotoxic	Brain-derived neurotrophic factor (BDNF) deletion in HD	BDNF is required for survival and dendritic growth of MSNs preferentially in indirect pathway.
		N-methyl D-aspartate receptor subtype 2B (NR2B) predominant expression in MSNs	High expression of NR2B in MSNs may promote NMDA excitotoxicity.
		Sensitivity to glutamate	MSNs are more sensitive to kainite, α-amino-3-hydroxy-5-methyl-4-isoxazolepropionic acid receptor (AMPA), N-methyl D-aspartate receptor (NMDA), and group 1 metabotropic glutamate receptor (mGluR) agonists than cholinergic interneurons.
		Increased NR2B containing extrasynaptic NMDA receptors	Extrasynaptic NMDA receptors increase the toxic mHtt and cause dysregulation in p38 mitogen-activated protein kinase-cAMP response element binding protein (MAPK-CREB) signaling.
		Requirement of higher energy in MSNs	Susceptibility for mitochondrial dysfunction induced by mutant-Htt.
		Expression level of superoxide dismutases (SODs)	MSNs contain low level of SODs, which indicates the vulnerability against oxidative stress.
	Neuroprotective	Increased expression of parvalbumin (PVA), calretinin, and calcium binding proteins	Calcium-buffering effect over excessive calcium-induced excitotoxicity.
		PDE10A deletion	Activation of extracellular signal-regulated kinase (ERK), CREB and predominant activation of D_2_R indirect pathway.
PVAs	Neurotoxic	BDNF deletion	Blockade of tropomyosin receptor kinase B-phospholipase Cγ (TrkB-PLCγ) pathway by BDNF deletion.
		AMPA receptors expression	Enrichment in Ca^2+^ permeable AMPA receptors induced calcium-induced excitotoxicity.
Interneurons	Neuroprotective	Optineurin expression	Optineurin is predominantly expressed in striatal interneurons and negatively regulates glutamate receptors via interaction with Htt.
Striosomes	Neurotoxic	Phosphodiesterase 10A (PDE-10A) decrease in cortical regions projecting to striosomes	PDE-10A decrease induces neurodegeneration in cortical neurons projecting to striosomes.
		Dopamine D_1_R	Enrichment of D_1_R in striosomes induces dopamine excitotoxicity.
		SOD2	Lower expression level of SOD2 in striosomes induces vulnerability against oxidative stress.
		Olfactory type G-protein α subunit (Gαolf)	Enrichment of Gαolf in striosomes induces D_1_R and A_2A_R mediated neurotoxicity (decreased level of Rhes increases A_2A_R/cAMP/protein kinase A (PKA) activity).
Matrix	Neuroprotective	Cyclin-dependent kinase 5 (CDK5)	Matrix enrich protein CDK5-pY15 induces phosphorylation of Htt which results in decrease of toxic effects against mutant-Htt.
		Decrease of calcium diacylglycerol guanine nucleotide exchange factor 1 (CalDAG-GEF1)	Matrix and MSNs predominant expression pattern of CalDAG-GEF1 protects them from mutant-Htt induced toxicity.
		Dopamine D_2_R	Enrichment of D_2_R in matrix is protective against dopamine excitotoxicity.
		Neuropeptide Y (NPY)	Enrichment of NPY in matrix is protective against glutamate excitotoxicity and microglial activation.
		28 kDa calbindin (Calbindin-D28K)	Enrichment of Calbindin-D28K in matrix is protective against excessive Ca^2+^ influx by calcium-buffering.
		Post synaptic density 95 kDa (PSD-95)	Matrix enrich protein PSD-95 is protective against glutamatergic or dopaminergic excitotoxicity.

## References

[1] Albin R.L., Tagle D.A. (1995). Genetics and molecular biology of huntington’s disease. Trends Neurosci..

[2] Roos R.A. (2010). Huntington’s disease: A clinical review. Orphanet J. Rare Dis..

[3] Rub U., Vonsattel J.P., Heinsen H., Korf H.W. (2015). The neuropathology of Huntington’S disease: Classical findings, recent developments and correlation to functional neuroanatomy. Adv. Anat. Embryol. Cell Biol..

[4] Khalil B., El Fissi N., Aouane A., Cabirol-Pol M.J., Rival T., Lievens J.C. (2015). Pink1-induced mitophagy promotes neuroprotection in huntington’s disease. Cell Death Dis..

[5] Goto S., Hirano A., Rojas-Corona R.R. (1989). An immunohistochemical investigation of the human neostriatum in huntington’s disease. Ann. Neurol..

[6] Augood S.J., Faull R.L., Love D.R., Emson P.C. (1996). Reduction in enkephalin and substance P messenger RNA in the striatum of early grade huntington’s disease: A detailed cellular in situ hybridization study. Neuroscience.

[7] Morton A.J., Nicholson L.F., Faull R.L. (1993). Compartmental loss of nadph diaphorase in the neuropil of the human striatum in huntington’s disease. Neuroscience.

[8] Hedreen J.C., Folstein S.E. (1995). Early loss of neostriatal striosome neurons in huntington’s disease. J. Neuropathol. Exp. Neurol..

[9] Tippett L.J., Waldvogel H.J., Thomas S.J., Hogg V.M., van Roon-Mom W., Synek B.J., Graybiel A.M., Faull R.L. (2007). Striosomes and mood dysfunction in huntington’s disease. Brain.

[10] Faull R.L., Waldvogel H.J., Nicholson L.F., Synek B.J. (1993). The distribution of gabaa-benzodiazepine receptors in the basal ganglia in huntington’s disease and in the quinolinic acid-lesioned rat. Prog. Brain Res..

[11] Seto-Ohshima A., Emson P.C., Lawson E., Mountjoy C.Q., Carrasco L.H. (1988). Loss of matrix calcium-binding protein-containing neurons in huntington’s disease. Lancet.

[12] Ferrante R.J., Kowall N.W., Beal M.F., Martin J.B., Bird E.D., Richardson E.P. (1987). Morphologic and histochemical characteristics of a spared subset of striatal neurons in huntington’s disease. J. Neuropathol. Exp. Neurol..

[13] Reiner A., Albin R.L., Anderson K.D., D’Amato C.J., Penney J.B., Young A.B. (1988). Differential loss of striatal projection neurons in huntington disease. Proc. Natl. Acad. Sci. USA.

[14] Albin R.L., Reiner A., Anderson K.D., Penney J.B., Young A.B. (1990). Striatal and nigral neuron subpopulations in rigid huntington’s disease: Implications for the functional anatomy of chorea and rigidity-akinesia. Ann. Neurol..

[15] Albin R.L., Qin Y., Young A.B., Penney J.B., Chesselet M.F. (1991). Preproenkephalin messenger rna-containing neurons in striatum of patients with symptomatic and presymptomatic huntington’s disease: An in situ hybridization study. Ann. Neurol..

[16] Albin R.L., Reiner A., Anderson K.D., Dure L.S., Handelin B., Balfour R., Whetsell W.O., Penney J.B., Young A.B. (1992). Preferential loss of striato-external pallidal projection neurons in presymptomatic huntington’s disease. Ann. Neurol..

[17] Albin R.L. (1995). Selective neurodegeneration in huntington’s disease. Ann. Neurol..

[18] Reiner A., Shelby E., Wang H., Demarch Z., Deng Y., Guley N.H., Hogg V., Roxburgh R., Tippett L.J., Waldvogel H.J. (2013). Striatal parvalbuminergic neurons are lost in huntington’s disease: Implications for dystonia. Mov. Disord..

[19] Goto S., Nagahiro S., Kaji R. (2010). Striosome-Matrix Pathology of Dystonias: A New Hypothesis for Dystonia Genesis.

[20] Graveland G.A., DiFiglia M. (1985). The frequency and distribution of medium-sized neurons with indented nuclei in the primate and rodent neostriatum. Brain Res..

[21] Cicchetti F., Prensa L., Wu Y., Parent A. (2000). Chemical anatomy of striatal interneurons in normal individuals and in patients with huntington’s disease. Brain Res. Brain Res. Rev..

[22] Tepper J.M., Wilson C.J., Koos T. (2008). Feedforward and feedback inhibition in neostriatal gabaergic spiny neurons. Brain Res. Rev..

[23] Tepper J.M., Tecuapetla F., Koos T., Ibanez-Sandoval O. (2010). Heterogeneity and diversity of striatal gabaergic interneurons. Front. Neuroanat..

[24] Ibanez-Sandoval O., Tecuapetla F., Unal B., Shah F., Koos T., Tepper J.M. (2010). Electrophysiological and morphological characteristics and synaptic connectivity of tyrosine hydroxylase-expressing neurons in adult mouse striatum. J. Neurosci..

[25] Koos T., Tepper J.M. (2002). Dual cholinergic control of fast-spiking interneurons in the neostriatum. J. Neurosci..

[26] Centonze D., Gubellini P., Pisani A., Bernardi G., Calabresi P. (2003). Dopamine, acetylcholine and nitric oxide systems interact to induce corticostriatal synaptic plasticity. Rev. Neurosci..

[27] Holt D.J., Hersh L.B., Saper C.B. (1996). Cholinergic innervation in the human striatum: A three-compartment model. Neuroscience.

[28] Bolam J.P. (2010). Microcircuits of the Striatum.

[29] Goto S., Morigaki R., Okita S., Nagahiro S., Kaji R. (2015). Development of a highly sensitive immunohistochemical method to detect neurochemical molecules in formalin-fixed and paraffin-embedded tissues from autopsied human brains. Front. Neuroanat..

[30] Alexander G.E., Crutcher M.D. (1990). Functional architecture of basal ganglia circuits: Neural substrates of parallel processing. Trends Neurosci..

[31] Gerfen C.R. (1992). The neostriatal mosaic: Multiple levels of compartmental organization. Trends Neurosci..

[32] Kreitzer A.C., Malenka R.C. (2008). Striatal plasticity and basal ganglia circuit function. Neuron.

[33] Nishi A., Kuroiwa M., Miller D.B., O’Callaghan J.P., Bateup H.S., Shuto T., Sotogaku N., Fukuda T., Heintz N., Greengard P. (2008). Distinct roles of PDE4 and PDE10A in the regulation of camp/pka signaling in the striatum. J. Neurosci..

[34] Nishi A., Kuroiwa M., Shuto T. (2011). Mechanisms for the modulation of dopamine D(1) receptor signaling in striatal neurons. Front. Neuroanat..

[35] Wilson C.J. (2007). Gabaergic inhibition in the neostriatum. Prog. Brain Res..

[36] Tepper J.M., Koos T., Wilson C.J. (2004). Gabaergic microcircuits in the neostriatum. Trends Neurosci..

[37] Graybiel A.M., Ragsdale C.W. (1978). Histochemically distinct compartments in the striatum of human, monkeys, and cat demonstrated by acetylthiocholinesterase staining. Proc. Natl. Acad. Sci. USA.

[38] Graybiel A.M. (1990). Neurotransmitters and neuromodulators in the basal ganglia. Trends Neurosci..

[39] Mikula S., Parrish S.K., Trimmer J.S., Jones E.G. (2009). Complete 3D visualization of primate striosomes by KCHIP1 immunostaining. J. Comp. Neurol..

[40] Moon Edley S., Herkenham M. (1984). Comparative development of striatal opiate receptors and dopamine revealed by autoradiography and histofluorescence. Brain Res..

[41] Fishell G., van der Kooy D. (1987). Pattern formation in the striatum: Developmental changes in the distribution of striatonigral neurons. J. Neurosci..

[42] Johnston J.G., Gerfen C.R., Haber S.N., van der Kooy D. (1990). Mechanisms of striatal pattern formation: Conservation of mammalian compartmentalization. Brain Res. Dev. Brain Res..

[43] van der Kooy D., Fishell G. (1987). Neuronal birthdate underlies the development of striatal compartments. Brain Res..

[44] Krushel L.A., Connolly J.A., van der Kooy D. (1989). Pattern formation in the mammalian forebrain: Patch neurons from the rat striatum selectively reassociate in vitro. Brain Res. Dev. Brain Res..

[45] Krushel L.A., Fishell G., Van der Kooy D. (1995). Pattern formation in the mammalian forebrain: Striatal patch and matrix neurons intermix prior to compartment formation. Eur. J. Neurosci..

[46] Holt D.J., Graybiel A.M., Saper C.B. (1997). Neurochemical architecture of the human striatum. J. Comp. Neurol..

[47] Gerfen C.R. (1985). The neostriatal mosaic. I. Compartmental organization of projections from the striatum to the substantia nigra in the rat. J. Comp. Neurol..

[48] Jimenez-Castellanos J., Graybiel A.M. (1989). Evidence that histochemically distinct zones of the primate substantia nigra pars compacta are related to patterned distributions of nigrostriatal projection neurons and striatonigral fibers. Exp. Brain Res..

[49] Fujiyama F., Sohn J., Nakano T., Furuta T., Nakamura K.C., Matsuda W., Kaneko T. (2011). Exclusive and common targets of neostriatofugal projections of rat striosome neurons: A single neuron-tracing study using a viral vector. Eur. J. Neurosci..

[50] Ragsdale C.W., Graybiel A.M. (1991). Compartmental organization of the thalamostriatal connection in the cat. J. Comp. Neurol..

[51] Christoph G.R., Leonzio R.J., Wilcox K.S. (1986). Stimulation of the lateral habenula inhibits dopamine-containing neurons in the substantia nigra and ventral tegmental area of the rat. J. Neurosci..

[52] Ji H., Shepard P.D. (2007). Lateral habenula stimulation inhibits rat midbrain dopamine neurons through a GABA(A) receptor-mediated mechanism. J. Neurosci..

[53] Matsumoto M., Hikosaka O. (2007). Lateral habenula as a source of negative reward signals in dopamine neurons. Nature.

[54] Bromberg-Martin E.S., Matsumoto M., Nakahara H., Hikosaka O. (2010). Multiple timescales of memory in lateral habenula and dopamine neurons. Neuron.

[55] Gerfen C.R. (1992). The neostriatal mosaic: Multiple levels of compartmental organization in the basal ganglia. Annu. Rev. Neurosci..

[56] Donoghue J.P., Herkenham M. (1986). Neostriatal projections from individual cortical fields conform to histochemically distinct striatal compartments in the rat. Brain Res..

[57] Ragsdale C.W., Graybiel A.M. (1988). Fibers from the basolateral nucleus of the amygdala selectively innervate striosomes in the caudate nucleus of the cat. J. Comp. Neurol..

[58] Bayer S.A. (1990). Neurogenetic patterns in the medial limbic cortex of the rat related to anatomical connections with the thalamus and striatum. Exp. Neurol..

[59] Flaherty A.W., Graybiel A.M. (1991). Corticostriatal transformations in the primate somatosensory system. Projections from physiologically mapped body-part representations. J. Neurophysiol..

[60] Flaherty A.W., Graybiel A.M. (1993). Two input systems for body representations in the primate striatal matrix: Experimental evidence in the squirrel monkey. J. Neurosci..

[61] Eblen F., Graybiel A.M. (1995). Highly restricted origin of prefrontal cortical inputs to striosomes in the macaque monkey. J. Neurosci..

[62] Levesque M., Parent A. (1998). Axonal arborization of corticostriatal and corticothalamic fibers arising from prelimbic cortex in the rat. Cereb. Cortex.

[63] Wang H., Pickel V.M. (1998). Dendritic spines containing mu-opioid receptors in rat striatal patches receive asymmetric synapses from prefrontal corticostriatal afferents. J. Comp. Neurol..

[64] Penny G.R., Wilson C.J., Kitai S.T. (1988). Relationship of the axonal and dendritic geometry of spiny projection neurons to the compartmental organization of the neostriatum. J. Comp. Neurol..

[65] Walker R.H., Arbuthnott G.W., Baughman R.W., Graybiel A.M. (1993). Dendritic domains of medium spiny neurons in the primate striatum: Relationships to striosomal borders. J. Comp. Neurol..

[66] Graybiel A.M., Baughman R.W., Eckenstein F. (1986). Cholinergic neuropil of the striatum observes striosomal boundaries. Nature.

[67] Graybiel A.M., Aosaki T., Flaherty A.W., Kimura M. (1994). The basal ganglia and adaptive motor control. Science.

[68] Aosaki T., Tsubokawa H., Ishida A., Watanabe K., Graybiel A.M., Kimura M. (1994). Responses of tonically active neurons in the primate’s striatum undergo systematic changes during behavioral sensorimotor conditioning. J. Neurosci..

[69] Aosaki T., Kimura M., Graybiel A.M. (1995). Temporal and spatial characteristics of tonically active neurons of the primate’s striatum. J. Neurophysiol..

[70] Miura M., Masuda M., Aosaki T. (2008). Roles of micro-opioid receptors in gabaergic synaptic transmission in the striosome and matrix compartments of the striatum. Mol. Neurobiol..

[71] Aosaki T., Miura M., Suzuki T., Nishimura K., Masuda M. (2010). Acetylcholine-dopamine balance hypothesis in the striatum: An update. Geriatr. Gerontol. Int..

[72] Amemori K., Gibb L.G., Graybiel A.M. (2011). Shifting responsibly: The importance of striatal modularity to reinforcement learning in uncertain environments. Front. Hum. Neurosci..

[73] Yamanaka K., Hori Y., Minamimoto T., Yamada H., Matsumoto N., Enomoto K., Aosaki T., Graybiel A.M., Kimura M. (2017). Roles of centromedian parafascicular nuclei of thalamus and cholinergic interneurons in the dorsal striatum in associative learning of environmental events. J. Neural Transm. (Vienna).

[74] Graybiel A.M., Canales J.J., Capper-Loup C. (2000). Levodopa-induced dyskinesias and dopamine-dependent stereotypies: A new hypothesis. Trends Neurosci..

[75] Graybiel A.M. (2008). Habits, rituals, and the evaluative brain. Annu. Rev. Neurosci..

[76] Steiner H., Gerfen C.R. (1998). Role of dynorphin and enkephalin in the regulation of striatal output pathways and behavior. Exp. Brain Res..

[77] Capper-Loup C., Canales J.J., Kadaba N., Graybiel A.M. (2002). Concurrent activation of dopamine D1 and D2 receptors is required to evoke neural and behavioral phenotypes of cocaine sensitization. J. Neurosci..

[78] Saka E., Goodrich C., Harlan P., Madras B.K., Graybiel A.M. (2004). Repetitive behaviors in monkeys are linked to specific striatal activation patterns. J. Neurosci..

[79] Goto S., Lee L.V., Munoz E.L., Tooyama I., Tamiya G., Makino S., Ando S., Dantes M.B., Yamada K., Matsumoto S. (2005). Functional anatomy of the basal ganglia in x-linked recessive dystonia-parkinsonism. Ann. Neurol..

[80] Smith K.S., Virkud A., Deisseroth K., Graybiel A.M. (2012). Reversible online control of habitual behavior by optogenetic perturbation of medial prefrontal cortex. Proc. Natl. Acad. Sci. USA.

[81] Aosaki T., Kawaguchi Y. (1996). Actions of substance p on rat neostriatal neurons in vitro. J. Neurosci..

[82] Cragg S.J. (2006). Meaningful silences: How dopamine listens to the ach pause. Trends Neurosci..

[83] Aosaki T., Graybiel A.M., Kimura M. (1994). Effect of the nigrostriatal dopamine system on acquired neural responses in the striatum of behaving monkeys. Science.

[84] Wang Z., Kai L., Day M., Ronesi J., Yin H.H., Ding J., Tkatch T., Lovinger D.M., Surmeier D.J. (2006). Dopaminergic control of corticostriatal long-term synaptic depression in medium spiny neurons is mediated by cholinergic interneurons. Neuron.

[85] Shen W., Flajolet M., Greengard P., Surmeier D.J. (2008). Dichotomous dopaminergic control of striatal synaptic plasticity. Science.

[86] Surmeier D.J., Ding J., Day M., Wang Z., Shen W. (2007). D1 and D2 dopamine-receptor modulation of striatal glutamatergic signaling in striatal medium spiny neurons. Trends Neurosci..

[87] Morigaki R., Okita S., Goto S. (2017). Dopamine-induced changes in galphaolf protein levels in striatonigral and striatopallidal medium spiny neurons underlie the genesis of l-dopa-induced dyskinesia in parkinsonian mice. Front. Cell. Neurosci..

[88] Sato K., Sumi-Ichinose C., Kaji R., Ikemoto K., Nomura T., Nagatsu I., Ichinose H., Ito M., Sako W., Nagahiro S. (2008). Differential involvement of striosome and matrix dopamine systems in a transgenic model of dopa-responsive dystonia. Proc. Natl. Acad. Sci. USA.

[89] Crittenden J.R., Dunn D.E., Merali F.I., Woodman B., Yim M., Borkowska A.E., Frosch M.P., Bates G.P., Housman D.E., Lo D.C. (2010). Caldag-gefi down-regulation in the striatum as a neuroprotective change in huntington’s disease. Hum. Mol. Genet..

[90] Imarisio S., Carmichael J., Korolchuk V., Chen C.W., Saiki S., Rose C., Krishna G., Davies J.E., Ttofi E., Underwood B.R. (2008). Huntington’s disease: From pathology and genetics to potential therapies. Biochem. J..

[91] Ratovitski T., Chighladze E., Arbez N., Boronina T., Herbrich S., Cole R.N., Ross C.A. (2012). Huntingtin protein interactions altered by polyglutamine expansion as determined by quantitative proteomic analysis. Cell Cycle.

[92] Rigamonti D., Bauer J.H., De-Fraja C., Conti L., Sipione S., Sciorati C., Clementi E., Hackam A., Hayden M.R., Li Y. (2000). Wild-type huntingtin protects from apoptosis upstream of caspase-3. J. Neurosci..

[93] Rigamonti D., Sipione S., Goffredo D., Zuccato C., Fossale E., Cattaneo E. (2001). Huntingtin’s neuroprotective activity occurs via inhibition of procaspase-9 processing. J. Biol. Chem..

[94] Ho L.W., Brown R., Maxwell M., Wyttenbach A., Rubinsztein D.C. (2001). Wild type huntingtin reduces the cellular toxicity of mutant huntingtin in mammalian cell models of huntington’s disease. J. Med. Genet..

[95] Duyao M.P., Auerbach A.B., Ryan A., Persichetti F., Barnes G.T., McNeil S.M., Ge P., Vonsattel J.P., Gusella J.F., Joyner A.L. (1995). Inactivation of the mouse huntington’s disease gene homolog hdh. Science.

[96] Nasir J., Floresco S.B., O’Kusky J.R., Diewert V.M., Richman J.M., Zeisler J., Borowski A., Marth J.D., Phillips A.G., Hayden M.R. (1995). Targeted disruption of the huntington’s disease gene results in embryonic lethality and behavioral and morphological changes in heterozygotes. Cell.

[97] Zeitlin S., Liu J.P., Chapman D.L., Papaioannou V.E., Efstratiadis A. (1995). Increased apoptosis and early embryonic lethality in mice nullizygous for the huntington’s disease gene homologue. Nat. Genet..

[98] Gervais F.G., Singaraja R., Xanthoudakis S., Gutekunst C.A., Leavitt B.R., Metzler M., Hackam A.S., Tam J., Vaillancourt J.P., Houtzager V. (2002). Recruitment and activation of caspase-8 by the huntingtin-interacting protein hip-1 and a novel partner hippi. Nat. Cell Biol..

[99] Zhang Y., Leavitt B.R., van Raamsdonk J.M., Dragatsis I., Goldowitz D., MacDonald M.E., Hayden M.R., Friedlander R.M. (2006). Huntingtin inhibits caspase-3 activation. EMBO J..

[100] Zuccato C., Tartari M., Crotti A., Goffredo D., Valenza M., Conti L., Cataudella T., Leavitt B.R., Hayden M.R., Timmusk T. (2003). Huntingtin interacts with rest/nrsf to modulate the transcription of nrse-controlled neuronal genes. Nat. Genet..

[101] Gauthier L.R., Charrin B.C., Borrell-Pages M., Dompierre J.P., Rangone H., Cordelieres F.P., De Mey J., MacDonald M.E., Lessmann V., Humbert S. (2004). Huntingtin controls neurotrophic support and survival of neurons by enhancing bdnf vesicular transport along microtubules. Cell.

[102] Gunawardena S., Her L.S., Brusch R.G., Laymon R.A., Niesman I.R., Gordesky-Gold B., Sintasath L., Bonini N.M., Goldstein L.S. (2003). Disruption of axonal transport by loss of huntingtin or expression of pathogenic polyq proteins in drosophila. Neuron.

[103] Trushina E., Dyer R.B., Badger J.D., Ure D., Eide L., Tran D.D., Vrieze B.T., Legendre-Guillemin V., McPherson P.S., Mandavilli B.S. (2004). Mutant huntingtin impairs axonal trafficking in mammalian neurons in vivo and in vitro. Mol. Cell Biol..

[104] McGuire J.R., Rong J., Li S.H., Li X.J. (2006). Interaction of huntingtin-associated protein-1 with kinesin light chain: Implications in intracellular trafficking in neurons. J. Biol. Chem..

[105] Caviston J.P., Ross J.L., Antony S.M., Tokito M., Holzbaur E.L. (2007). Huntingtin facilitates dynein/dynactin-mediated vesicle transport. Proc. Natl. Acad. Sci. USA.

[106] Zuccato C., Cattaneo E. (2007). Role of brain-derived neurotrophic factor in huntington’s disease. Prog. Neurobiol..

[107] Berghuis P., Agerman K., Dobszay M.B., Minichiello L., Harkany T., Ernfors P. (2006). Brain-derived neurotrophic factor selectively regulates dendritogenesis of parvalbumin-containing interneurons in the main olfactory bulb through the plcgamma pathway. J. Neurobiol..

[108] Morfini G., Pigino G., Brady S.T. (2005). Polyglutamine expansion diseases: Failing to deliver. Trends Mol. Med..

[109] Orr H.T., Zoghbi H.Y. (2007). Trinucleotide repeat disorders. Annu. Rev. Neurosci..

[110] Han I., You Y., Kordower J.H., Brady S.T., Morfini G.A. (2010). Differential vulnerability of neurons in huntington’s disease: The role of cell type-specific features. J. Neurochem..

[111] Davies S.W., Turmaine M., Cozens B.A., DiFiglia M., Sharp A.H., Ross C.A., Scherzinger E., Wanker E.E., Mangiarini L., Bates G.P. (1997). Formation of neuronal intranuclear inclusions underlies the neurological dysfunction in mice transgenic for the hd mutation. Cell.

[112] Schilling G., Becher M.W., Sharp A.H., Jinnah H.A., Duan K., Kotzuk J.A., Slunt H.H., Ratovitski T., Cooper J.K., Jenkins N.A. (1999). Intranuclear inclusions and neuritic aggregates in transgenic mice expressing a mutant N-terminal fragment of huntingtin. Hum. Mol. Genet..

[113] Palfi S., Brouillet E., Jarraya B., Bloch J., Jan C., Shin M., Conde F., Li X.J., Aebischer P., Hantraye P. (2007). Expression of mutated huntingtin fragment in the putamen is sufficient to produce abnormal movement in non-human primates. Mol. Ther..

[114] Graham R.K., Deng Y., Slow E.J., Haigh B., Bissada N., Lu G., Pearson J., Shehadeh J., Bertram L., Murphy Z. (2006). Cleavage at the caspase-6 site is required for neuronal dysfunction and degeneration due to mutant huntingtin. Cell.

[115] Humbert S., Bryson E.A., Cordelieres F.P., Connors N.C., Datta S.R., Finkbeiner S., Greenberg M.E., Saudou F. (2002). The igf-1/akt pathway is neuroprotective in huntington’s disease and involves huntingtin phosphorylation by akt. Dev. Cell.

[116] Luo S., Vacher C., Davies J.E., Rubinsztein D.C. (2005). Cdk5 phosphorylation of huntingtin reduces its cleavage by caspases: Implications for mutant huntingtin toxicity. J. Cell. Biol..

[117] Schilling B., Gafni J., Torcassi C., Cong X., Row R.H., LaFevre-Bernt M.A., Cusack M.P., Ratovitski T., Hirschhorn R., Ross C.A. (2006). Huntingtin phosphorylation sites mapped by mass spectrometry. Modulation of cleavage and toxicity. J. Biol. Chem..

[118] Seong I.S., Ivanova E., Lee J.M., Choo Y.S., Fossale E., Anderson M., Gusella J.F., Laramie J.M., Myers R.H., Lesort M. (2005). Hd cag repeat implicates a dominant property of huntingtin in mitochondrial energy metabolism. Hum. Mol. Genet..

[119] Panov A.V., Gutekunst C.A., Leavitt B.R., Hayden M.R., Burke J.R., Strittmatter W.J., Greenamyre J.T. (2002). Early mitochondrial calcium defects in huntington’s disease are a direct effect of polyglutamines. Nat. Neurosci..

[120] Reddy P.H., Mao P., Manczak M. (2009). Mitochondrial structural and functional dynamics in huntington’s disease. Brain Res. Rev..

[121] Orr A.L., Li S., Wang C.E., Li H., Wang J., Rong J., Xu X., Mastroberardino P.G., Greenamyre J.T., Li X.J. (2008). N-terminal mutant huntingtin associates with mitochondria and impairs mitochondrial trafficking. J. Neurosci..

[122] Li X.J., Orr A.L., Li S. (2010). Impaired mitochondrial trafficking in huntington’s disease. Biochim. Biophys. Acta.

[123] Calabresi P., De Murtas M., Pisani A., Stefani A., Sancesario G., Mercuri N.B., Bernardi G. (1995). Vulnerability of medium spiny striatal neurons to glutamate: Role of Na+/K+ atpase. Eur. J. Neurosci..

[124] Roy O.W., Cohen N.R., Nicoll J.A. (2005). Pathophysiology of dementias and implications for therapy. Indian J. Pathol. Microbiol..

[125] Morfini G.A., Burns M., Binder L.I., Kanaan N.M., LaPointe N., Bosco D.A., Brown R.H., Brown H., Tiwari A., Hayward L. (2009). Axonal transport defects in neurodegenerative diseases. J. Neurosci..

[126] Morfini G.A., You Y.M., Pollema S.L., Kaminska A., Liu K., Yoshioka K., Bjorkblom B., Coffey E.T., Bagnato C., Han D. (2009). Pathogenic huntingtin inhibits fast axonal transport by activating JNK3 and phosphorylating kinesin. Nat. Neurosci..

[127] Benn C.L., Sun T., Sadri-Vakili G., McFarland K.N., DiRocco D.P., Yohrling G.J., Clark T.W., Bouzou B., Cha J.H. (2008). Huntingtin modulates transcription, occupies gene promoters in vivo, and binds directly to DNA in a polyglutamine-dependent manner. J. Neurosci..

[128] Pagano G., Niccolini F., Politis M. (2016). Current status of pet imaging in huntington’s disease. Eur. J. Nucl. Med. Mol. Imaging.

[129] Wilson H., De Micco R., Niccolini F., Politis M. (2017). Molecular imaging markers to track huntington’s disease pathology. Front. Neurol..

[130] Xie Z., Adamowicz W.O., Eldred W.D., Jakowski A.B., Kleiman R.J., Morton D.G., Stephenson D.T., Strick C.A., Williams R.D., Menniti F.S. (2006). Cellular and subcellular localization of pde10a, a striatum-enriched phosphodiesterase. Neuroscience.

[131] Sano H., Nagai Y., Miyakawa T., Shigemoto R., Yokoi M. (2008). Increased social interaction in mice deficient of the striatal medium spiny neuron-specific phosphodiesterase 10A2. J. Neurochem..

[132] Miller S., Hill Della Puppa G., Reidling J., Marcora E., Thompson L.M., Treanor J. (2014). Comparison of phosphodiesterase 10A, dopamine receptors D1 and D2 and dopamine transporter ligand binding in the striatum of the R6/2 and bachd mouse models of huntington’s disease. J. Huntingt. Dis..

[133] Ooms M., Rietjens R., Rangarajan J.R., Vunckx K., Valdeolivas S., Maes F., Himmelreich U., Fernandez-Ruiz J., Bormans G., Van Laere K. (2014). Early decrease of type 1 cannabinoid receptor binding and phosphodiesterase 10A activity in vivo in R6/2 huntington mice. Neurobiol. Aging.

[134] Fujishige K., Kotera J., Michibata H., Yuasa K., Takebayashi S., Okumura K., Omori K. (1999). Cloning and characterization of a novel human phosphodiesterase that hydrolyzes both camp and cgmp (PDE10A). J. Biol. Chem..

[135] Niccolini F., Haider S., Reis Marques T., Muhlert N., Tziortzi A.C., Searle G.E., Natesan S., Piccini P., Kapur S., Rabiner E.A. (2015). Altered pde10a expression detectable early before symptomatic onset in huntington’s disease. Brain.

[136] Wilson H., Niccolini F., Haider S., Marques T.R., Pagano G., Coello C., Natesan S., Kapur S., Rabiner E.A., Gunn R.N. (2016). Loss of extra-striatal phosphodiesterase 10A expression in early premanifest huntington’s disease gene carriers. J. Neurol. Sci..

[137] Russell D.S., Barret O., Jennings D.L., Friedman J.H., Tamagnan G.D., Thomae D., Alagille D., Morley T.J., Papin C., Papapetropoulos S. (2014). The phosphodiesterase 10 positron emission tomography tracer, [18F]mni-659, as a novel biomarker for early huntington disease. JAMA Neurol..

[138] Giampa C., Patassini S., Borreca A., Laurenti D., Marullo F., Bernardi G., Menniti F.S., Fusco F.R. (2009). Phosphodiesterase 10 inhibition reduces striatal excitotoxicity in the quinolinic acid model of huntington’s disease. Neurobiol. Dis..

[139] Giampa C., Laurenti D., Anzilotti S., Bernardi G., Menniti F.S., Fusco F.R. (2010). Inhibition of the striatal specific phosphodiesterase pde10A ameliorates striatal and cortical pathology in R6/2 mouse model of huntington’s disease. PLoS ONE.

[140] Threlfell S., Sammut S., Menniti F.S., Schmidt C.J., West A.R. (2009). Inhibition of phosphodiesterase 10A increases the responsiveness of striatal projection neurons to cortical stimulation. J. Pharmacol. Exp. Ther..

[141] Tai Y.F., Pavese N., Gerhard A., Tabrizi S.J., Barker R.A., Brooks D.J., Piccini P. (2007). Microglial activation in presymptomatic huntington’s disease gene carriers. Brain.

[142] Politis M., Pavese N., Tai Y.F., Kiferle L., Mason S.L., Brooks D.J., Tabrizi S.J., Barker R.A., Piccini P. (2011). Microglial activation in regions related to cognitive function predicts disease onset in huntington’s disease: A multimodal imaging study. Hum. Brain Mapp..

[143] Politis M., Lahiri N., Niccolini F., Su P., Wu K., Giannetti P., Scahill R.I., Turkheimer F.E., Tabrizi S.J., Piccini P. (2015). Increased central microglial activation associated with peripheral cytokine levels in premanifest huntington’s disease gene carriers. Neurobiol. Dis..

[144] Andrews T.C., Weeks R.A., Turjanski N., Gunn R.N., Watkins L.H., Sahakian B., Hodges J.R., Rosser A.E., Wood N.W., Brooks D.J. (1999). Huntington’s disease progression. Pet and clinical observations. Brain.

[145] Van Oostrom J.C., Maguire R.P., Verschuuren-Bemelmans C.C., Veenma-van der Duin L., Pruim J., Roos R.A., Leenders K.L. (2005). Striatal dopamine D2 receptors, metabolism, and volume in preclinical huntington disease. Neurology.

[146] Antonini A., Leenders K.L., Spiegel R., Meier D., Vontobel P., Weigell-Weber M., Sanchez-Pernaute R., de Yebenez J.G., Boesiger P., Weindl A. (1996). Striatal glucose metabolism and dopamine D2 receptor binding in asymptomatic gene carriers and patients with huntington’s disease. Brain.

[147] Kassubek J., Bernhard Landwehrmeyer G., Ecker D., Juengling F.D., Muche R., Schuller S., Weindl A., Peinemann A. (2004). Global cerebral atrophy in early stages of huntington’s disease: Quantitative mri study. Neuroreport.

[148] Douaud G., Gaura V., Ribeiro M.J., Lethimonnier F., Maroy R., Verny C., Krystkowiak P., Damier P., Bachoud-Levi A.C., Hantraye P. (2006). Distribution of grey matter atrophy in huntington’s disease patients: A combined roi-based and voxel-based morphometric study. Neuroimage.

[149] Sano H., Yasoshima Y., Matsushita N., Kaneko T., Kohno K., Pastan I., Kobayashi K. (2003). Conditional ablation of striatal neuronal types containing dopamine D2 receptor disturbs coordination of basal ganglia function. J. Neurosci..

[150] Li S.H., Schilling G., Young W.S., Li X.J., Margolis R.L., Stine O.C., Wagster M.V., Abbott M.H., Franz M.L., Ranen N.G. (1993). Huntington’s disease gene (IT15) is widely expressed in human and rat tissues. Neuron.

[151] Mestre T.A., Sampaio C. (2017). Huntington disease: Linking pathogenesis to the development of experimental therapeutics. Curr. Neurol. Neurosci. Rep..

[152] Shelbourne P.F., Keller-McGandy C., Bi W.L., Yoon S.R., Dubeau L., Veitch N.J., Vonsattel J.P., Wexler N.S., Group U.S.-V.C.R., Arnheim N. (2007). Triplet repeat mutation length gains correlate with cell-type specific vulnerability in huntington disease brain. Hum. Mol. Genet..

[153] Kennedy L., Evans E., Chen C.M., Craven L., Detloff P.J., Ennis M., Shelbourne P.F. (2003). Dramatic tissue-specific mutation length increases are an early molecular event in huntington disease pathogenesis. Hum. Mol. Genet..

[154] Furtado S., Suchowersky O., Rewcastle B., Graham L., Klimek M.L., Garber A. (1996). Relationship between trinucleotide repeats and neuropathological changes in huntington’s disease. Ann. Neurol..

[155] Zuccato C., Ciammola A., Rigamonti D., Leavitt B.R., Goffredo D., Conti L., MacDonald M.E., Friedlander R.M., Silani V., Hayden M.R. (2001). Loss of huntingtin-mediated bdnf gene transcription in huntington’s disease. Science.

[156] Szebenyi G., Morfini G.A., Babcock A., Gould M., Selkoe K., Stenoien D.L., Young M., Faber P.W., MacDonald M.E., McPhaul M.J. (2003). Neuropathogenic forms of huntingtin and androgen receptor inhibit fast axonal transport. Neuron.

[157] Her L.S., Goldstein L.S. (2008). Enhanced sensitivity of striatal neurons to axonal transport defects induced by mutant huntingtin. J. Neurosci..

[158] Rauskolb S., Zagrebelsky M., Dreznjak A., Deogracias R., Matsumoto T., Wiese S., Erne B., Sendtner M., Schaeren-Wiemers N., Korte M. (2010). Global deprivation of brain-derived neurotrophic factor in the cns reveals an area-specific requirement for dendritic growth. J. Neurosci..

[159] Canals J.M., Pineda J.R., Torres-Peraza J.F., Bosch M., Martin-Ibanez R., Munoz M.T., Mengod G., Ernfors P., Alberch J. (2004). Brain-derived neurotrophic factor regulates the onset and severity of motor dysfunction associated with enkephalinergic neuronal degeneration in huntington’s disease. J. Neurosci..

[160] Subramaniam S., Sixt K.M., Barrow R., Snyder S.H. (2009). Rhes, a striatal specific protein, mediates mutant-huntingtin cytotoxicity. Science.

[161] Ross C.A., Tabrizi S.J. (2011). Huntington’s disease: From molecular pathogenesis to clinical treatment. Lancet Neurol..

[162] Steffan J.S., Agrawal N., Pallos J., Rockabrand E., Trotman L.C., Slepko N., Illes K., Lukacsovich T., Zhu Y.Z., Cattaneo E. (2004). Sumo modification of huntingtin and huntington’s disease pathology. Science.

[163] Baiamonte B.A., Lee F.A., Brewer S.T., Spano D., LaHoste G.J. (2013). Attenuation of rhes activity significantly delays the appearance of behavioral symptoms in a mouse model of huntington’s disease. PLoS ONE.

[164] Mealer R.G., Subramaniam S., Snyder S.H. (2013). Rhes deletion is neuroprotective in the 3-nitropropionic acid model of huntington’s disease. J. Neurosci..

[165] Mealer R.G., Murray A.J., Shahani N., Subramaniam S., Snyder S.H. (2014). Rhes, a striatal-selective protein implicated in huntington disease, binds beclin-1 and activates autophagy. J. Biol. Chem..

[166] Li X.J., Li S. (2011). Proteasomal dysfunction in aging and huntington disease. Neurobiol. Dis..

[167] Hodges A., Strand A.D., Aragaki A.K., Kuhn A., Sengstag T., Hughes G., Elliston L.A., Hartog C., Goldstein D.R., Thu D. (2006). Regional and cellular gene expression changes in human huntington’s disease brain. Hum. Mol. Genet..

[168] Lee J.H., Tecedor L., Chen Y.H., Monteys A.M., Sowada M.J., Thompson L.M., Davidson B.L. (2015). Reinstating aberrant mTORC1 activity in huntington’s disease mice improves disease phenotypes. Neuron.

[169] Lee J.H., Sowada M.J., Boudreau R.L., Aerts A.M., Thedens D.R., Nopoulos P., Davidson B.L. (2014). Rhes suppression enhances disease phenotypes in huntington’s disease mice. J. Huntingt. Dis..

[170] Ferrante R.J., Kowall N.W., Beal M.F., Richardson E.P., Bird E.D., Martin J.B. (1985). Selective sparing of a class of striatal neurons in huntington’s disease. Science.

[171] Ferrante R.J., Beal M.F., Kowall N.W., Richardson E.P., Martin J.B. (1987). Sparing of acetylcholinesterase-containing striatal neurons in huntington’s disease. Brain Res..

[172] DiFiglia M., Sapp E., Chase K., Schwarz C., Meloni A., Young C., Martin E., Vonsattel J.P., Carraway R., Reeves S.A. (1995). Huntingtin is a cytoplasmic protein associated with vesicles in human and rat brain neurons. Neuron.

[173] Sharp A.H., Loev S.J., Schilling G., Li S.H., Li X.J., Bao J., Wagster M.V., Kotzuk J.A., Steiner J.P., Lo A. (1995). Widespread expression of huntington’s disease gene (IT15) protein product. Neuron.

[174] Bhide P.G., Day M., Sapp E., Schwarz C., Sheth A., Kim J., Young A.B., Penney J., Golden J., Aronin N. (1996). Expression of normal and mutant huntingtin in the developing brain. J. Neurosci..

[175] Gourfinkel-An I., Cancel G., Trottier Y., Devys D., Tora L., Lutz Y., Imbert G., Saudou F., Stevanin G., Agid Y. (1997). Differential distribution of the normal and mutated forms of huntingtin in the human brain. Ann. Neurol..

[176] Landwehrmeyer G.B., McNeil S.M., Dure L.S.t., Ge P., Aizawa H., Huang Q., Ambrose C.M., Duyao M.P., Bird E.D., Bonilla E. (1995). Huntington’s disease gene: Regional and cellular expression in brain of normal and affected individuals. Ann. Neurol..

[177] Schilling G., Sharp A.H., Loev S.J., Wagster M.V., Li S.H., Stine O.C., Ross C.A. (1995). Expression of the huntington’s disease (IT15) protein product in hd patients. Hum. Mol. Genet..

[178] Trottier Y., Devys D., Imbert G., Saudou F., An I., Lutz Y., Weber C., Agid Y., Hirsch E.C., Mandel J.L. (1995). Cellular localization of the huntington’s disease protein and discrimination of the normal and mutated form. Nat. Genet..

[179] Sapp E., Schwarz C., Chase K., Bhide P.G., Young A.B., Penney J., Vonsattel J.P., Aronin N., DiFiglia M. (1997). Huntingtin localization in brains of normal and huntington’s disease patients. Ann. Neurol..

[180] Senut M.C., Suhr S.T., Kaspar B., Gage F.H. (2000). Intraneuronal aggregate formation and cell death after viral expression of expanded polyglutamine tracts in the adult rat brain. J. Neurosci..

[181] De Almeida L.P., Ross C.A., Zala D., Aebischer P., Deglon N. (2002). Lentiviral-mediated delivery of mutant huntingtin in the striatum of rats induces a selective neuropathology modulated by polyglutamine repeat size, huntingtin expression levels, and protein length. J. Neurosci..

[182] Beal M.F., Kowall N.W., Ellison D.W., Mazurek M.F., Swartz K.J., Martin J.B. (1986). Replication of the neurochemical characteristics of huntington’s disease by quinolinic acid. Nature.

[183] Calabresi P., Pisani A., Mercuri N.B., Bernardi G. (1996). The corticostriatal projection: From synaptic plasticity to dysfunctions of the basal ganglia. Trends Neurosci..

[184] Calabresi P., Centonze D., Pisani A., Sancesario G., Gubellini P., Marfia G.A., Bernardi G. (1998). Striatal spiny neurons and cholinergic interneurons express differential ionotropic glutamatergic responses and vulnerability: Implications for ischemia and huntington’s disease. Ann. Neurol..

[185] Calabresi P., Centonze D., Pisani A., Bernardi G. (1999). Metabotropic glutamate receptors and cell-type-specific vulnerability in the striatum: Implication for ischemia and huntington’s disease. Exp. Neurol..

[186] Chesselet M.F., Gonzales C., Lin C.S., Polsky K., Jin B.K. (1990). Ischemic damage in the striatum of adult gerbils: Relative sparing of somatostatinergic and cholinergic interneurons contrasts with loss of efferent neurons. Exp. Neurol..

[187] DiFiglia M. (1990). Excitotoxic injury of the neostriatum: A model for huntington’s disease. Trends Neurosci..

[188] Goto S., Kawarai T., Morigaki R., Okita S., Koizumi H., Nagahiro S., Munoz E.L., Lee L.V., Kaji R. (2013). Defects in the striatal neuropeptide y system in x-linked dystonia-parkinsonism. Brain.

[189] Wagster M.V., Hedreen J.C., Peyser C.E., Folstein S.E., Ross C.A. (1994). Selective loss of [3H]kainic acid and [3H]AMPA binding in layer vi of frontal cortex in huntington’s disease. Exp. Neurol..

[190] Young A.B., Greenamyre J.T., Hollingsworth Z., Albin R., D’Amato C., Shoulson I., Penney J.B. (1988). Nmda receptor losses in putamen from patients with huntington’s disease. Science.

[191] Cha J.H., Kosinski C.M., Kerner J.A., Alsdorf S.A., Mangiarini L., Davies S.W., Penney J.B., Bates G.P., Young A.B. (1998). Altered brain neurotransmitter receptors in transgenic mice expressing a portion of an abnormal human huntington disease gene. Proc. Natl. Acad. Sci. USA.

[192] Cha J.H., Frey A.S., Alsdorf S.A., Kerner J.A., Kosinski C.M., Mangiarini L., Penney J.B., Davies S.W., Bates G.P., Young A.B. (1999). Altered neurotransmitter receptor expression in transgenic mouse models of huntington’s disease. Philos. Trans. R. Soc. Lond. B Biol. Sci..

[193] Nicniocaill B., Haraldsson B., Hansson O., O’Connor W.T., Brundin P. (2001). Altered striatal amino acid neurotransmitter release monitored using microdialysis in r6/1 huntington transgenic mice. Eur. J. Neurosci..

[194] Li H., Wyman T., Yu Z.X., Li S.H., Li X.J. (2003). Abnormal association of mutant huntingtin with synaptic vesicles inhibits glutamate release. Hum. Mol. Genet..

[195] Levine M.S., Klapstein G.J., Koppel A., Gruen E., Cepeda C., Vargas M.E., Jokel E.S., Carpenter E.M., Zanjani H., Hurst R.S. (1999). Enhanced sensitivity to n-methyl-d-aspartate receptor activation in transgenic and knockin mouse models of huntington’s disease. J. Neurosci. Res..

[196] Cepeda C., Ariano M.A., Calvert C.R., Flores-Hernandez J., Chandler S.H., Leavitt B.R., Hayden M.R., Levine M.S. (2001). Nmda receptor function in mouse models of huntington disease. J. Neurosci. Res..

[197] Starling A.J., Andre V.M., Cepeda C., de Lima M., Chandler S.H., Levine M.S. (2005). Alterations in N-methyl-D-aspartate receptor sensitivity and magnesium blockade occur early in development in the R6/2 mouse model of huntington’s disease. J. Neurosci. Res..

[198] Rigby M., Le Bourdelles B., Heavens R.P., Kelly S., Smith D., Butler A., Hammans R., Hills R., Xuereb J.H., Hill R.G. (1996). The messenger rnas for the N-methyl-D-aspartate receptor subunits show region-specific expression of different subunit composition in the human brain. Neuroscience.

[199] Landwehrmeyer G.B., Standaert D.G., Testa C.M., Penney J.B., Young A.B. (1995). Nmda receptor subunit mrna expression by projection neurons and interneurons in rat striatum. J. Neurosci..

[200] Kuppenbender K.D., Standaert D.G., Feuerstein T.J., Penney J.B., Young A.B., Landwehrmeyer G.B. (2000). Expression of nmda receptor subunit mrnas in neurochemically identified projection and interneurons in the human striatum. J. Comp. Neurol..

[201] Schoepfer R., Monyer H., Sommer B., Wisden W., Sprengel R., Kuner T., Lomeli H., Herb A., Kohler M., Burnashev N. (1994). Molecular biology of glutamate receptors. Prog. Neurobiol..

[202] Okamoto S., Pouladi M.A., Talantova M., Yao D., Xia P., Ehrnhoefer D.E., Zaidi R., Clemente A., Kaul M., Graham R.K. (2009). Balance between synaptic versus extrasynaptic nmda receptor activity influences inclusions and neurotoxicity of mutant huntingtin. Nat. Med..

[203] Milnerwood A.J., Gladding C.M., Pouladi M.A., Kaufman A.M., Hines R.M., Boyd J.D., Ko R.W., Vasuta O.C., Graham R.K., Hayden M.R. (2010). Early increase in extrasynaptic nmda receptor signaling and expression contributes to phenotype onset in huntington’s disease mice. Neuron.

[204] Milnerwood A.J., Raymond L.A. (2010). Early synaptic pathophysiology in neurodegeneration: Insights from huntington’s disease. Trends Neurosci..

[205] Dau A., Gladding C.M., Sepers M.D., Raymond L.A. (2014). Chronic blockade of extrasynaptic nmda receptors ameliorates synaptic dysfunction and pro-death signaling in huntington disease transgenic mice. Neurobiol. Dis..

[206] Gerfen C.R., Baimbridge K.G., Miller J.J. (1985). The neostriatal mosaic: Compartmental distribution of calcium-binding protein and parvalbumin in the basal ganglia of the rat and monkey. Proc. Natl. Acad. Sci. USA.

[207] Huang Q., Zhou D., Sapp E., Aizawa H., Ge P., Bird E.D., Vonsattel J.P., DiFiglia M. (1995). Quinolinic acid-induced increases in calbindin D28K immunoreactivity in rat striatal neurons in vivo and in vitro mimic the pattern seen in huntington’s disease. Neuroscience.

[208] Sun Z., Wang H.B., Deng Y.P., Lei W.L., Xie J.P., Meade C.A., Del Mar N., Goldowitz D., Reiner A. (2005). Increased calbindin-D28K immunoreactivity in striatal projection neurons of R6/2 huntington’s disease transgenic mice. Neurobiol. Dis..

[209] Deng Y.P., Shelby E., Reiner A.J. (2010). Immunohistochemical localization of ampa-type glutamate receptor subunits in the striatum of rhesus monkey. Brain Res..

[210] Okita S., Morigaki R., Koizumi H., Kaji R., Nagahiro S., Goto S. (2012). Cell type-specific localization of optineurin in the striatal neurons of mice: Implications for neuronal vulnerability in huntington’s disease. Neuroscience.

[211] Sun Y., Savanenin A., Reddy P.H., Liu Y.F. (2001). Polyglutamine-expanded huntingtin promotes sensitization of N-methyl-D-aspartate receptors via post-synaptic density 95. J. Biol. Chem..

[212] Anborgh P.H., Godin C., Pampillo M., Dhami G.K., Dale L.B., Cregan S.P., Truant R., Ferguson S.S. (2005). Inhibition of metabotropic glutamate receptor signaling by the huntingtin-binding protein optineurin. J. Biol. Chem..

[213] Gu M., Gash M.T., Mann V.M., Javoy-Agid F., Cooper J.M., Schapira A.H. (1996). Mitochondrial defect in huntington’s disease caudate nucleus. Ann. Neurol..

[214] Wilson C.J., Kawaguchi Y. (1996). The origins of two-state spontaneous membrane potential fluctuations of neostriatal spiny neurons. J. Neurosci..

[215] Lee J.M., Ivanova E.V., Seong I.S., Cashorali T., Kohane I., Gusella J.F., MacDonald M.E. (2007). Unbiased gene expression analysis implicates the huntingtin polyglutamine tract in extra-mitochondrial energy metabolism. PLoS Genet..

[216] Medina L., Figueredo-Cardenas G., Reiner A. (1996). Differential abundance of superoxide dismutase in interneurons versus projection neurons and in matrix versus striosome neurons in monkey striatum. Brain Res..

[217] Johri A., Beal M.F. (2012). Antioxidants in huntington’s disease. Biochim. Biophys. Acta.

[218] Ferrante R.J., Kowall N.W. (1987). Tyrosine hydroxylase-like immunoreactivity is distributed in the matrix compartment of normal human and huntington’s disease striatum. Brain Res..

[219] Lee J.M., Ramos E.M., Lee J.H., Gillis T., Mysore J.S., Hayden M.R., Warby S.C., Morrison P., Nance M., Ross C.A. (2012). Cag repeat expansion in huntington disease determines age at onset in a fully dominant fashion. Neurology.

[220] Keum J.W., Shin A., Gillis T., Mysore J.S., Abu Elneel K., Lucente D., Hadzi T., Holmans P., Jones L., Orth M. (2016). The htt cag-expansion mutation determines age at death but not disease duration in huntington disease. Am. J. Hum. Genet..

[221] Burke R.E., Baimbridge K.G. (1993). Relative loss of the striatal striosome compartment, defined by calbindin-D28K immunostaining, following developmental hypoxic-ischemic injury. Neuroscience.

[222] Fuchs T., Saunders-Pullman R., Masuho I., Luciano M.S., Raymond D., Factor S., Lang A.E., Liang T.W., Trosch R.M., White S. (2013). Mutations in gnal cause primary torsion dystonia. Nat. Genet..

[223] Sato K., Kaji R., Matsumoto S., Nagahiro S., Goto S. (2007). Compartmental loss of striatal medium spiny neurons in multiple system atrophy of parkinsonian type. Mov. Disord..

[224] Lawhorn C., Smith D.M., Brown L.L. (2008). Striosome-matrix pathology and motor deficits in the YAC128 mouse model of huntington’s disease. Neurobiol. Dis..

[225] Crittenden J.R., Graybiel A.M. (2011). Basal ganglia disorders associated with imbalances in the striatal striosome and matrix compartments. Front. Neuroanat..

[226] Schwab L.C., Garas S.N., Drouin-Ouellet J., Mason S.L., Stott S.R., Barker R.A. (2015). Dopamine and huntington’s disease. Expert Rev. Neurother..

[227] Andre V.M., Cepeda C., Levine M.S. (2010). Dopamine and glutamate in huntington’s disease: A balancing act. CNS Neurosci. Ther..

[228] Spokes E.G. (1980). Neurochemical alterations in huntington’s chorea: A study of post-mortem brain tissue. Brain.

[229] Garrett M.C., Soares-da-Silva P. (1992). Increased cerebrospinal fluid dopamine and 3,4-dihydroxyphenylacetic acid levels in huntington’s disease: Evidence for an overactive dopaminergic brain transmission. J. Neurochem..

[230] Yohrling G.J., Jiang G.C., DeJohn M.M., Miller D.W., Young A.B., Vrana K.E., Cha J.H. (2003). Analysis of cellular, transgenic and human models of huntington’s disease reveals tyrosine hydroxylase alterations and substantia nigra neuropathology. Brain Res. Mol. Brain Res..

[231] Jakel R.J., Maragos W.F. (2000). Neuronal cell death in huntington’s disease: A potential role for dopamine. Trends Neurosci..

[232] Hickey M.A., Reynolds G.P., Morton A.J. (2002). The role of dopamine in motor symptoms in the R6/2 transgenic mouse model of huntington’s disease. J. Neurochem..

[233] Reynolds G.P., Garrett N.J. (1986). Striatal dopamine and homovanillic acid in huntington’s disease. J. Neural Transm..

[234] Bozzi Y., Borrelli E. (2006). Dopamine in neurotoxicity and neuroprotection: What do D2 receptors have to do with it?. Trends Neurosci..

[235] Sako W., Morigaki R., Nagahiro S., Kaji R., Goto S. (2010). Olfactory type g-protein alpha subunit in striosome-matrix dopamine systems in adult mice. Neuroscience.

[236] Thapliyal A., Bannister R.A., Hanks C., Adams B.A. (2008). The monomeric g proteins AGS1 and rhes selectively influence galphai-dependent signaling to modulate N-type (CAV2.2) calcium channels. Am. J. Physiol. Cell Physiol..

[237] Harrison L.M., He Y. (2011). Rhes and AGS1/Dexras1 affect signaling by dopamine D1 receptors through adenylyl cyclase. J. Neurosci. Res..

[238] Vargiu P., De Abajo R., Garcia-Ranea J.A., Valencia A., Santisteban P., Crespo P., Bernal J. (2004). The small gtp-binding protein, rhes, regulates signal transduction from g protein-coupled receptors. Oncogene.

[239] Errico F., Santini E., Migliarini S., Borgkvist A., Centonze D., Nasti V., Carta M., De Chiara V., Prosperetti C., Spano D. (2008). The gtp-binding protein rhes modulates dopamine signalling in striatal medium spiny neurons. Mol. Cell Neurosci..

[240] Ghiglieri V., Napolitano F., Pelosi B., Schepisi C., Migliarini S., Di Maio A., Pendolino V., Mancini M., Sciamanna G., Vitucci D. (2015). Rhes influences striatal cAMP/PKA-dependent signaling and synaptic plasticity in a gender-sensitive fashion. Sci. Rep..

[241] Stockwell J., Jakova E., Cayabyab F.S. (2017). Adenosine A1 and A2A receptors in the brain: Current research and their role in neurodegeneration. Molecules.

[242] Dawbarn D., De Quidt M.E., Emson P.C. (1985). Survival of basal ganglia neuropeptide y-somatostatin neurones in huntington’s disease. Brain Res..

[243] Decressac M., Mattsson B., Bjorklund A. (2012). Comparison of the behavioural and histological characteristics of the 6-ohda and alpha-synuclein rat models of parkinson’s disease. Exp. Neurol..

[244] Beal M.F. (1998). Mitochondrial dysfunction in neurodegenerative diseases. Biochim. Biophys. Acta.

[245] Bezprozvanny I., Hayden M.R. (2004). Deranged neuronal calcium signaling and huntington disease. Biochem. Biophys. Res. Commun..

[246] Tang T.S., Chen X., Liu J., Bezprozvanny I. (2007). Dopaminergic signaling and striatal neurodegeneration in huntington’s disease. J. Neurosci..

[247] Morigaki R., Goto S. (2015). Postsynaptic density protein 95 in the striosome and matrix compartments of the human neostriatum. Front. Neuroanat..

[248] Greengard P. (2001). The neurobiology of dopamine signaling. Biosci. Rep..

[249] Morigaki R., Sako W., Okita S., Kasahara J., Yokoyama H., Nagahiro S., Kaji R., Goto S. (2011). Cyclin-dependent kinase 5 with phosphorylation of tyrosine 15 residue is enriched in striatal matrix compartment in adult mice. Neuroscience.

